# Advances in Polyvinyl Alcohol-Based Membranes for Fuel Cells: A Comprehensive Review on Types, Synthesis, Modifications, and Performance Optimization

**DOI:** 10.3390/polym16131775

**Published:** 2024-06-23

**Authors:** Chandra Mouli R. Madhuranthakam, Weam S. K. Abudaqqa, Michael Fowler

**Affiliations:** 1 Chemical Engineering Department, Abu Dhabi University, Abu Dhabi P.O. Box 59911, United Arab Emirates; weama.research@adu.ac.ae; 2 Chemical Engineering Department, University of Waterloo, Waterloo, ON N2L 3G5, Canada; mfowler@uwaterloo.ca

**Keywords:** polyvinyl alcohol (PVA), fuel cells, nanocomposites, hybrid membranes, polymers

## Abstract

Fuel cell technology is at the forefront of sustainable energy solutions, and polyvinyl alcohol (PVA) membranes play an important role in improving performance. This article thoroughly investigates the various varieties of PVA membranes, their production processes, and the numerous modification tactics used to solve inherent problems. Various methods were investigated, including chemical changes, composite blending, and the introduction of nanocomposites. The factors impacting PVA membranes, such as proton conductivity, thermal stability, and selectivity, were investigated to provide comprehensive knowledge. By combining various research threads, this review aims to completely investigate the current state of PVA membranes in fuel cell applications, providing significant insights for both academic researchers and industry practitioners interested in efficient and sustainable energy conversion technologies. The transition from traditional materials such as Nafion to PVA membranes has been prompted by limitations associated with the former, such as complex synthesis procedures, reduced ionic conductivity at elevated temperatures, and prohibitively high costs, which have hampered their widespread adoption. As a result, modern research efforts are increasingly focused on the creation of alternative membranes that can compete with conventional technical efficacy and economic viability in the context of fuel cell technologies.

## 1. Introduction

The exponential growth of the world’s population, combined with increased anthropogenic activities, has resulted in a steady increase in energy and water consumption. Addressing this difficulty can be accomplished through the use of membrane technology, which is critical in fuel cells for energy generation [1,2,3]. A membrane acts as a semipermeable barrier layer, allowing certain entities to pass while preventing others. A membrane’s design is determined by various criteria, including the intended use, target species, process operating principles, feed stream or fluid composition, and desired product properties. Careful evaluation of these aspects is critical in selecting the membrane’s chemical functioning, shape, pore size, dimensions, and configuration. As a result, the preparation of a membrane, based on structural and functional criteria, requires the selection of appropriate polymers [4,5,6].

**Figure 1:**
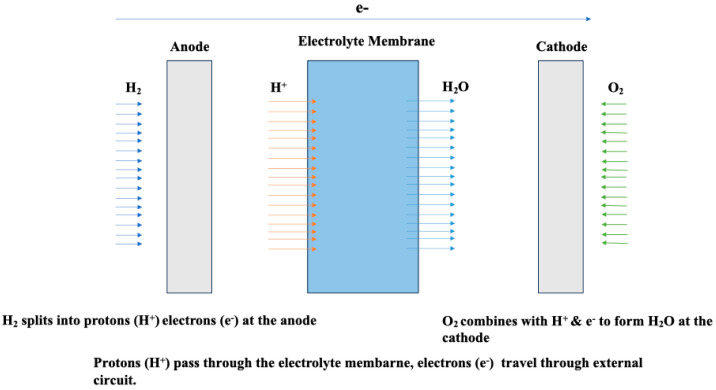
Two-dimensional diagram of a fuel cell membrane. Redrawn from [26].

As energy conversion devices, fuel cells continuously transform chemical energy from a fuel into electrical energy, as illustrated in Figure 1, provided both the fuel and oxidant are available [7]. In addition, fuel cells are electrochemical devices characterized by operational temperatures exceeding 1000 °C. They facilitate the conversion of chemical energy, sourced from elements such as oxygen, hydrogen, and natural gas, into electricity, water, and heat through catalytic processes [8]. In fact, the energy density of fuel cells surpasses that of conventional energy devices, which make them exhibit a notable suitability for prolonged consumption scenarios (as illustrated in Table 1). Consequently, the recognized advantages associated with fuel cells are expected to incentivize further advancements in fuel cell membrane technology.

**Table 1:** Comparative Analysis of Energy Systems and Technologies.

Device	Energy Density	Life Time	Benefits	Drawbacks	Refs.
Fuel Cell	Very high	5000–10,000 (hours)	Modular and compact design Exceptional efficiency Swift hydrogen refueling Negligible emissions	Prolonged cold start Cost-intensive Risks associated with hydrogen Elevated fuel expenses	[7,9,10,11]
Battery	High	4–6 (years)	Portable and rechargeable functionality Economical Well-established technology	Slow recharge rate Limited lifespan Battery preparation and recycling contribute to environmental pollution Flammable electrolyte	[12,13]
Supercapacitor	Very low	10–20 (years)	Prompt recharging and response	Short time energy storage High cost	[14,15,16]
Photovoltaic panel	Medium	25–30 (years)	Environmentally sustainable	Power output is intermittent Huge for light transport	[17,18]
Flywheels	High	5–10 (years)	Elevated power Environmentally sustainable	Slow charging Heavy weight	[19,20]
Superconducting magnetic energy storage system	Low	25–30 (years)	Elevated power output and performance Remarkable efficiency Environmentally conscious Rapid response time	Short-term energy storage High cost	[21,22,23]

The membranes employed in fuel cells are often developed using cationic or anionic functionalized polymers. With a fuel cell stack made up of 370 cells, the Toyota Mirai stands out as one of the first fuel cell cars. GORE-SELECTVR membrane technology is included in every one of these cells. The fuel cell stack has an estimated 9 m^2^ of total active area. Notably, the polymer electrolyte membrane (PEM), specifically the NafionTM N117-type membrane, carries a high price tag of USD 17,919 according to the NafionTM store. It is noteworthy that this expense amounts to about thirty-one percent of the car’s quoted selling price of USD 57,500 which highlights the high cost of these membranes [24,25].

Various polymers have been investigated for the fuel cell industry, ranging from laboratory to pilot sizes, such as perfluorosulfonic acid-based copolymers, sulfonated hydrocarbon polymers, sulfonated aromatic polymers, acid-doped polybenzimidazole (PBI), polyphosphazene, and a variety of quaternized polymers. Yet, several of these polymers are inefficient while remaining expensive [27,28,29,30]. In response, researchers have dedicated efforts to identify innovative polymers that can replace traditional ones, aiming to increase efficiency and lower costs in the advancement of membrane technology for sustainable energy generation. For this, poly(vinyl alcohol) (PVA) stands out as a promising choice among several alternative options. PVA is characterized by its abundant hydroxyl groups within its chemical structure and exhibits robust hydrogen bonding with water, resulting in excellent methanol resistance [31,32]. Owing to these beneficial characteristics, PVA has been thoroughly studied for the creation of membranes meant to be used in alkaline and direct methanol fuel cells and production of membranes that are surface-modified, chlorine-resistant, and antifouling materials in water treatment [33,34]. This review aims to provide a comprehensive overview of the types, synthesis methods, modifications, and performance optimizations of PVA-based membranes used in fuel cells. To ensure a thorough and relevant analysis, articles were selected through extensive searches in major scientific databases such as Google Scholar, ScienceDirect, and MDPI using keywords like “PVA membranes”, “fuel cells”, Nanocomposites”, “Hybrid Membranes”, and “Polymers”. The selection criteria focused on research articles, review papers, and significant patents published over the past decade (2014–2024), capturing recent advancements and trends. Topics were chosen for their importance to the field of PVA-based membranes for fuel cells, identified by examining the frequency and impact of specific topics in the literature and through expert consultations. The paper is structured to present a logical flow of information, beginning with a fundamental overview of PVA and its applications in different types of fuel cells, followed by synthesis methods, performance-influencing factors, and enhancements. The review concludes with discussions on challenges, future prospects, and a final summary. This approach not only synthesizes current knowledge and advancements but also identifies critical areas for future research. By addressing the synthesis, modifications, and performance optimization of PVA membranes, this article aims to provide a valuable resource for both academic and industrial researchers working towards more efficient and cost-effective fuel cell technologies, ultimately enhancing the practical applications of PVA-based membranes in fuel cells.

### Fundamental Overview of PVA

PVA is a notable example of a water-soluble, biodegradable polymer. PVA is not directly polymerized from the appropriate monomer, unlike the majority of vinyl polymers. Rather, a number of indirect techniques are used, like the alcoholysis of polyvinyl acetate (also referred to as saponification or hydrolysis) [35]. This conversion process can be facilitated through the use of potent acids or bases. As a polymeric material, PVA boasts exceptional properties that contribute to its widespread utilization across diverse industrial applications. Remarkable attributes include its excellent film-forming capacity, structural flexibility, hydrophilicity, and biocompatibility [36,37]. Consequently, PVA finds extensive use in various industrial domains, including but not limited to the recovery of acids and valuable metals from waste solutions, membrane fuel cell applications, biological and biomedical applications, catalysis in organic and inorganic syntheses, as well as pervaporation separation of liquid mixtures [38,39,40,41].

In fact, PVA is a copolymer with hydroxyl and acetyl units resulting from the partial hydrolysis of the parent polymer, vinyl acetate. As a result, polymers with more than 50% hydroxyl groups are classed as poly (vinyl alcohols), and polymers with more than 50% acetyl groups are labeled as poly (vinylacetates). The degree of hydrolysis (DH), defined as the percentage of

**Figure 2:**
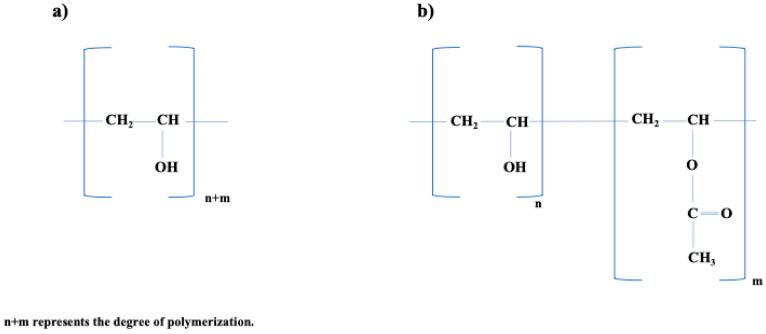
Chemical Structure of PVA. (**a**) Fully hydrolyzed PVA structure. (**b**) Partially hydrolyzed PVA. Redrawn from [44].

OH^−^-Groups in the final product, is critical in determining the characteristics of PVA [42]. Additionally, commercially accessible PVA has variable degrees of hydrolysis, which are commonly classified as 70–72%mol, 87–89%mol, or 99+%mol (completely hydrolyzed), as seen in Figure 2 [43].

The DH has a significant impact on PVA solubility, with fully hydrolyzed grades being more difficult to dissolve and often requiring hot water, while lower hydrolysis grades provide more viscous aqueous solutions. This solubility phenomena is linked to the creation of strong hydrogen intramolecular bonds in fully hydrolyzed grades as opposed to lesser hydrolysis counterparts [45,46]. Table 2 demonstrates the critical properties of PVA with their corresponding description.

## 2. Exploring the Role of PVA in Membrane Technology

**Figure 3:**
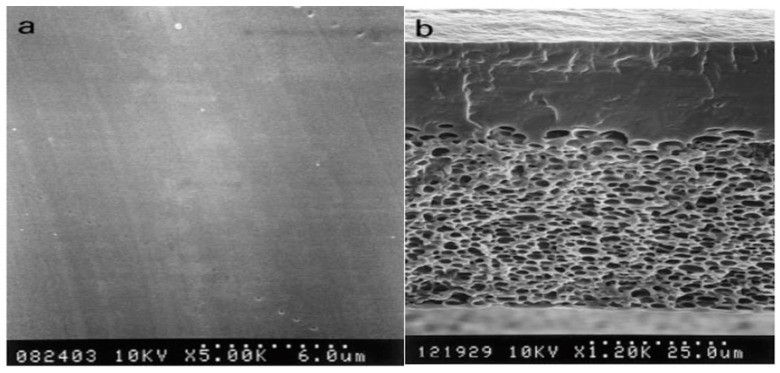
SEM Micrographs of PVA Membrane: (**a**) top surface and (**b**) cross-section. Reproduced with permission from [65].

In light of the enumerated advantages of PVA [60,61,62], membranes based on PVA exhibit significant characteristics such as excellent water absorption capacity, strong strength and flexibility, and reduced permeability to methanol. Figure 3 illustrates that the PVA membranes exhibit an asymmetric structure comprising a dense skin layer and a porous sublayer. The sublayer contains cellular morphologies encapsulated within the polymer matrix. The dense skin layer is primarily responsible for the selective permeation or rejection of solutes, while the porous sublayer provides mechanical support. However, the fundamental limitation stems from the lack of charged functional groups, such as sulfonic acid (SO_3_H) or carboxylic acid (COOH) groups, which results in low ionic conductivity in PVA membranes [63]. Integrating powerful ionic groups such as sulfonic, phosphonic, and quaternary ammonium ions as proton sources into the PVA matrix appears to be a promising option to overcome this constraint. This enhancement increases both the ionic conductivity and the hydrophilicity of PVA membranes [64].

**Figure 4:**
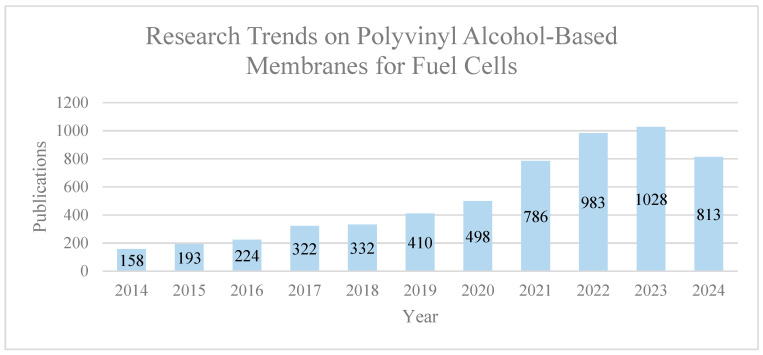
Research Trends in Publications on Polyvinyl Alcohol (PVA)-Based Membranes for Fuel Cells (2014–2024). Extracted from ScienceDirect on 1 June 2024.

Various membrane types have been systematically explored and regarded as promising fuel cell technologies. Notably, the application of PVA in direct methanol fuel cell (DMFC) technology has garnered attention. This interest stems from the high permeation selectivity exhibited by the proton-exchange membrane (PEM) made of PVA, particularly in enabling water passage while acting as a superior barrier against methanol [66]. Beyond PEM, the ongoing development of proton-conducting and methanol barrier membranes for DMFCs is recognized as a pioneering approach, offering potential improvements in proton conduction. This advancement aims to strike a balance between enhanced proton conduction and controlled methanol permeability [67]. Furthermore, the exploration of anion-exchange membranes (AEMs) for alkaline fuel cells stands as a significant avenue in fuel cell technology. In this context, the selection of suitable polymers for AEMs is pivotal, as it can result in membranes with excellent hydroxide ion (OH^−^) conductivity, chemical and thermal stability, strength, flexibility, low gas permeability, minimal water drag, cost effectiveness, and widespread availability [68]. Figure 4 illustrates the research trends in publications on PVA-based membranes for fuel cells over the past decade (2014–2024). The data indicate a notable increase in interest and research activity in this field, with a steady rise in the number of publications each year. Although 2024 has not yet concluded, the current trajectory suggests that the number of publications will continue to grow, reflecting the ongoing advancements and focus on PVA-based membranes in fuel cell technology. Analyzing these trends is critical for understanding the evolution and optimization of fuel cell technologies, as each advancement contributes to the overall progress in this area.

### 2.1. Hydrogen Fuel Cells by PVA–Proton-Exchange Membranes

**Figure 5:**
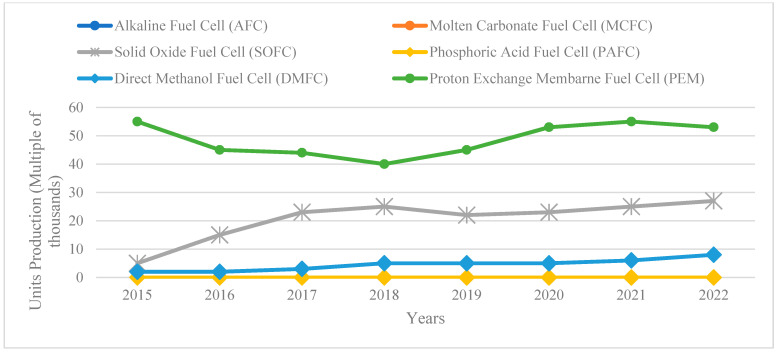
Progression of PEM Technologies. Extracted from [69,75].

As the most identifiable fuel cell type among others, Figure 5 illustrates the significant developments in PEMs over the past few years [69]. This fuel cell stands out for its lightweight construction, which allows it to run at a comparatively low temperature (80 °C) with a high efficiency (>60%). Moreover, the PEMs guarantee no emissions of greenhouse gases, and its byproducts are water or H_2_O, which are environmentally safe. This makes it an adaptable option for both heavy- and light-duty operations [70,71,72]. In the composition of a PEMs, essential components include end plates, current collectors, bipolar plates (BP), gaskets, gas diffusion layers (GDL), catalyst, and membrane. All of these parts operate in tandem to make the fuel cell function. Particularly, the end plates, equipped with unique flow channels like single serpentine, double serpentine, and four serpentine configurations, ensure structural integrity and prevent gas leakage into the environment [73,74].

**Figure 6:**
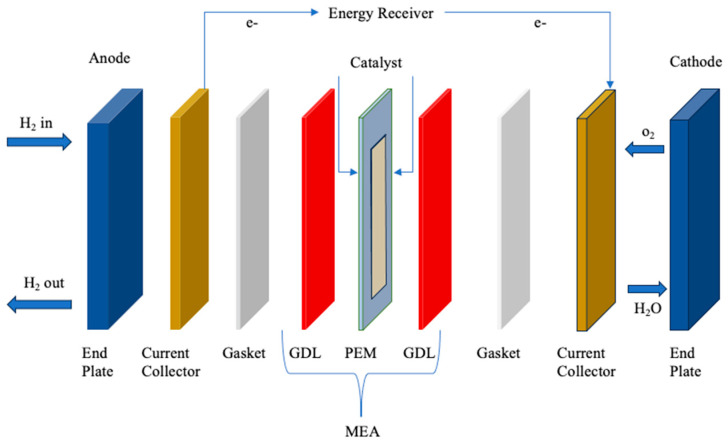
Schematic representation of PEM. Redrawn from [79].

In order to gather energy and transfer it from the fuel cell to the exterior, the current collector is essential. Meanwhile, the bipolar plate makes it easier to link two membrane electrode assemblies (MEAs), which raises the voltage and releases water [76], while the gaskets (composed of rubber) are used in fuel cells to stop reactant gasses from leaking out [77]. The MEA stands out as the primary component of proton-exchange membrane fuel cells (PEMFCs) (seen in Figure 6). Their fabrication can be achieved through various methods, including catalyst-coated membrane (CCM), catalyst-coated substrate (CCS), and catalyst-coated electrode (CCE) [78]. Each of these methods contributes to the overall functionality of the PEMFC, ensuring efficient energy conversion.

**Figure 7:**
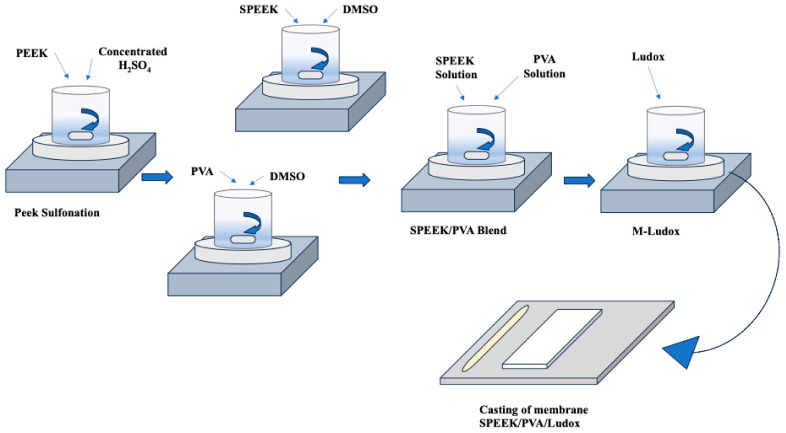
Solution Casting Method for Synthesizing SPEEK/PVA Blend Membranes with Colloidal Silica Additives. Adopted from [86].

Research has looked into the development of proton-exchange membranes (PEMs) using hydrocarbon-based polymers [80]. The motivation behind this investigation lies in the desire to reduce membrane costs, enhance membrane performance at elevated temperatures (as demonstrated in Table 3), and mitigate methanol permeability [81]. In particular, non-fluorinated hydrocarbon polymers that go beyond conventional choices have been used to create PEMs for H_2_/O_2_ fuel cells with the goal of reducing expenses and improving fuel cell efficiency. Among these alternatives, non-fluorinated hydrocarbon polymers—especially those derived from polyvinyl alcohol (PVA)—have shown to be cost-effective and simple to manufacture. For that, PVA-based polymers have attracted a lot of interest [82]. One approach that is seen to be promising is the integration of PVA-based polymers into Nafion ionomer and the creation of membranes with a low thickness [83]. Using this method, Molla et al. [84] pursued the fabrication of a nanofibrous mat with a thickness ranging from 19 to 60 μm. This was achieved by electrospinning PVA solution, followed by a series of chemical modifications involving 4-formyl-1,3-benzenedisulfonic acid and cross-linking with glutaraldehyde (GA). The resulting PVA mat underwent further enhancement by impregnation with a Nafion ionomer solution, leading to the creation of ultrathin Nafion/PVA composite membranes with thicknesses ranging from 20 to 33 μm. A membrane of notable thickness (19 μm) in this investigation showed a power density of 0.477 W cm^−2^ at 0.5 V and 960 mA cm^−2^. This power density was found to be similar to that of Nafion/polytetrafluoroethylene (PTFE) composite membranes, albeit marginally less than that of pristine Nafion membranes [85]. Furthermore, Yagizatli et al. [86] developed an inexpensive membrane with high proton conductivity and excellent fuel cell performance as a potential alternative to Nafion for PEMFCs. Sulfonated polyether ether ketone (SPEEK) and PVA blend membranes with colloidal silica additives (Ludox AS-40) were synthesized using the solution casting method (as represented in Figure 7). The membranes were evaluated for water uptake capacity, ion-exchange capacity, proton conductivity, and stability. At 80 °C, the water uptake capacities for 1%, 5%, and 10% Ludox-added SPEEK/PVA membranes were 14.73%, 15.17%, and 17.11%, respectively. Their proton conductivities were 0.25 S/cm, 0.56 S/cm, and 0.65 S/cm. The ion-exchange capacities were 1.41 meq/g, 1.63 meq/g, and 1.71 meq/g. The membranes exhibited excellent hydrolytic stability, retaining about 88% of their mass after 650 h. In oxidative stability tests, the 5% Ludox-additive membrane retained 91.6% of its total weight after 24 h. These findings indicate that the synthesized membranes are promising candidates for PEMFC applications due to their high proton conductivity and durability.

In another study by Muhmed et al. [87], a novel organic-inorganic nanocomposite membrane was prepared by incorporating sulfonated holey graphene oxide (SHGO) into a nanocrystalline cellulose/polyvinyl alcohol (NCC/PVA) matrix using the solution casting method. The introduction of sulfonic acid groups into graphene oxide (GO) resulted in sulfonated graphene oxide (SGO), and the hole effect on the graphitic plane of GO significantly improved the proton conductivity of the membranes. The highest proton conductivity of 1.1 × 10^−2^ S/cm was achieved by NCC/PVA-SHGO-1.0 at 80 °C under 100% relative humidity (RH). Mechanical and chemical stability tests also showed enhanced properties. However, hydrogen permeation increased slightly due to the hole effect. At 80 °C and 100% RH, NCC/PVA-SHGO-1.0 exhibited the highest power density (31.4 mW/cm^2^) and current density (60.2 mA/cm^2^), which were three times higher than those of pristine NCC/PVA. This study concluded that incorporating SHGO into NCC/PVA membranes significantly improves their properties, performance, and durability, making them potential candidates for PEMs.

**Table 3:** Temperature-Dependent Performance Analysis of various PEMFCs. Reproduced from [88,89,90,91,92,93,94,95,96,97,98].

Type of PEMFC	PEM	Water Uptake (%)	Thermal Stability T_m_ (°C)	Ion-Exchange Capacity (meq/g)	Proton Conductivity (S/cm)	Fuel Cell Performance (mV/cm^2^)
Low Temperature	PVA/SBA-15-propyl-SO_3_H	80 (25 °C)	150–185	-	0.006 (25 °C)	-
	PVA/SGO	58.3 (25 °C)	214–223	0.92 (25 °C)	0.050 (25 °C)	16.15 (30 °C)
	SPEEK/PVA/TEOS	65 (80 °C)	-	2.02 (25 °C)	0.084 (80 °C)	336 (80 °C)
	PVA/SSA	-	-	-	0.077 (70 °C)	99 (70 °C)
	PVA/PAMPS/ZIF	328 (25 °C)	<200	1.52 (25 °C)	0.134 (80 °C)	-
	25 kGy irradiation doses 10% PVA	-	>220	-	0.340 (25 °C)	-
	PVA/SSA/GO	-	325	-	0.003 (30 °C)	155.4 (23 °C)
	PVA/sulfonic acid	-	-	-	0.156 × 10^−3^ (25 °C)	-
High Temperature (>120 °C)	PVA/PAMPS/1.2.4-triazole	248 (25 °C)	<250	1.62 (25 °C)	0.002 (150 °C)	-
	H_3_PO_4_-imbibed PAM/PVA	-	100–200	-	0.053 (183 °C)	225 (183 °C)

### 2.2. PVA–Proton-Conducting and Methanol Barrier Membranes for Direct Methanol Cells

PVA-based PEMs for DMFC applications have progressed principally due to two key objectives (Table 4): (1) improving methanol barrier characteristics and (2) lowering membrane production costs. In DMFC, the migration of methanol from the anode to the cathode side causes a mixed potential at the cathode side, which contributes to reduced fuel cell performance and cell polarization [99]. Both DMFC and PEMFC use polymers as proton-conducting membranes. While the types of catalysts and fuels used for feeding vary, the overall design of DMFC and PEMFC is almost identical [100]. The electrolyte in DMFC is a polymer substance comparable to PEMFC, and methanol is directly used as fuel. In the DMFC [101], methanol is directly oxidized to carbon dioxide and protons at the anode according to Equations (1)–(3):

(1) $${CH}_{3}OH+H_{2}O\to {CO}_{2}+6e^{-}+6H^{+}$$

Followed by a reduction of oxygen, which occurs at the cathode using the following Equation (2):

(2) $$1.5O_{2}+6H^{+}+6e^{-}\to {3H}_{2}O$$

which leads to a net reaction through Equation (3) [102]:

(3) $$CH_{3}OH+1.5{CO}_{2}\to {CO}_{2}+{2H}_{2}O$$

The migration of methanol from the anode to the cathode via the PEM, also known as methanol crossover, is a major issue in DMFCs. The phenomenon increases the cathode’s overpotential, poisoning the cathode catalyst and significantly reducing fuel cell efficiency. As a result, the choice of a polymer matrix for the PEM in a DMFC system has been extensively studied in order to reduce methanol crossover and obtain high proton conductivity [103,104,105,106]. Consequently, numerous studies have investigated PVA-based PEMs because of PVA’s remarkable methanol barrier capabilities, which are related to its dense structure and enabled by strong intra- and intermolecular hydrogen bonds. PVA-based PEMs with semi-IPN (interpenetrating polymer network) structures were created by combining PVA with polyanions such as poly(acrylic acid) [107], poly(styrene sulfonic acid-co-maleic acid) [108,109], poly(2-acrylamido-2-methylpropane sulfonic acid) [110], and phosphotungstic acid [111]. Lue et al. [112] conducted a study reporting a methanol permeability of 3.57 × 10^−7^ cm^2^/s for a pure polyvinyl alcohol (PVA) membrane. Subsequent improvement in methanol resistivity was demonstrated through chemical cross-linking with sulfoacetic acid sodium salt and polyacrylic acid (PAA), as reported by Seeponkai et al. [113]. The methanol permeability was enhanced to 5.21 × 10^−9^ cm^2^/s with a 10wt.% sulfoacetic acid sodium salt-to-PVA weight ratio. The resulting sulfonated membrane exhibited an increase in proton conductivity (from 3.06 × 10^−3^ S/cm to 0.57 × 10^−3^ S/cm of pure PVA) and ion-exchange capacity (IEC) (from 0.462 to 0.092 meq/g of pure PVA). These findings suggest that chemically cross-linking pure PVA by introducing proton sources such as sulfoacetic acid sodium salt has the potential to address the intrinsic issue of low conductivity in pure PVA membranes and positions them as promising polymeric membranes for DMFC applications. Khalaf et al. [114] also found a PEM made of phosphorylated PVA and grafted cellulose acetate (CA) to have a low methanol permeability of 1.08 × 10^−10^ cm^2^/s. The results demonstrated a considerable improvement in proton conductivity, increasing from 0.035 to 0.05 S/cm at 25 and 70 °C, respectively, using 600 μL of OPA (O-phosphoric acid) and an ion-exchange capacity (IEC) of 2.1 meq/g using 400 μL of OPA at room temperature. The produced Ph-PVA/CA membranes improved important parameters such as proton conductivity and IEC while remaining cost-effective.

### 2.3. PVA–Anion-Exchange Membrane of Alkaline Fuel Cells

Alkaline fuel cells (AFCs) are electrochemical devices that transform chemical energy into electrical energy. It uses an aqueous solution of potassium hydroxide (KOH), the most conductive of all alkali metal hydroxides, as the electrolyte [115]. AFCs are the earliest type of fuel cell, and they have a long history of use in NASA’s Gemini space program [116,117]. The process involves the hydrogen ions present on the anode reacting with hydroxyl anions, resulting in the production of water and electrons. These electrons are subsequently transferred through an external circuit to the cathode, where oxygen engages with water, yielding hydroxyl ions. The overall reactions are expressed as follows in Equations (4)–(6):

(4) $$\mathrm{Anode}\;\mathrm{reaction}\;2H_{2}+4{OH}^{-}\to {4H}_{2}O+{4e}^{-}$$

(5) $$\mathrm{Cathode}\;\mathrm{reaction}\;O_{2}+{2H}_{2}O+{4e}^{-}\to 4{OH}^{-}$$

(6) $$\mathrm{Overall}\;\mathrm{reaction}\;2H_{2}+O_{2}\to {2H}_{2}O+electrical\;energy+heat$$

The key advantages of the alkaline fuel cell (AFC) are its comparatively low operating temperature requirements (60–70 °C) and less demanding surroundings for operation when compared to PEMFCs. Furthermore, the reaction kinetics are faster in a basic medium than in acidic circumstances, resulting in higher cell output voltages [115,118].

Anion-exchange membranes (AEMs) are classified into two types: homogeneous and heterogeneous. Homogeneous AEMs have a single-phase structure, with the ion-exchanging material incorporating covalently bound cationic functional charges into the polymer backbone. Homogeneous AEMs can be prepared using direct polymerization, which involves the polymerization or polycondensation of monomers containing cationic moieties; prefunctionalization, which involves casting a polymer solution with pre-existing cationic moieties into a film; or postfunctionalization, which involves the introduction of cationic moieties into a preformed polymer film [119,120]. On the contrary, heterogeneous AEMs are made up of an ion-exchanging substance and an inert matrix material. This category includes ion-solvating polymers such as PVA, which has been doped with conducting ion salt solutions like KOH [115,121,122,123]. In this context, polymers are primarily responsible for mechanical qualities. Heterogeneous AEMs also give rise to hybrid membranes, which are made up of both organic and inorganic segments. The organic compound, typically an ion-exchanging polymer, provides conducting properties, while the inorganic component, which includes alkoxysilane, oxides (e.g., SiO_2_, Al_2_O_3_, TiO_2_, and ZrO_2_), and nano-carbon materials such as graphene and CNT, improves mechanical, thermal, chemical, and ion-conducting properties [124]. This hybrid technique aims to synergize the beneficial characteristics of both organic and inorganic components, providing a comprehensive improvement in AEM performance across diverse dimensions.

**Figure 8:**
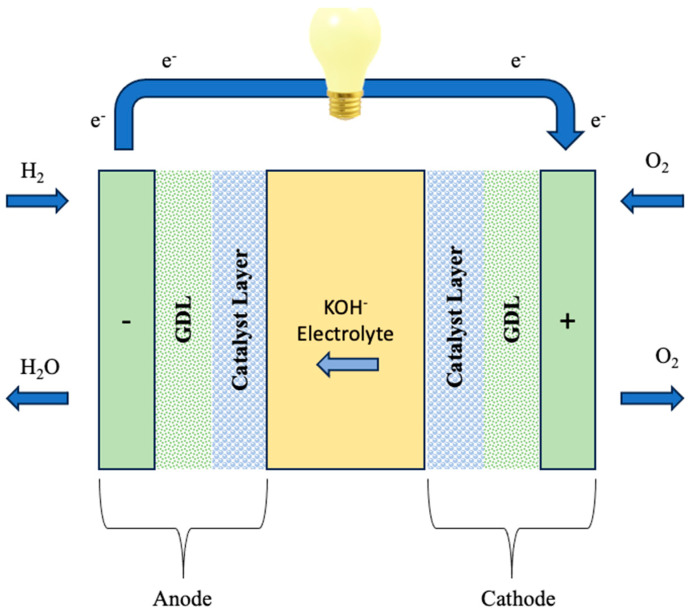
Schematic representation of AEMFC. Redrawn from [128].

A single alkaline exchange membrane fuel cell (AEMFC) contains necessary hardware components, such as a gasket that forms a seal around the membrane electrode assembly (MEA) to prevent gas leakage and protect the cell. The MEA includes the alkaline exchange membrane (AEM), the anode and cathode catalyst layers, and the cathode gas diffusion layer (GDL) (Figure 8). The use of an AEM creates an alkaline environment inside the fuel cell. In this scenario, the most prevalent anion species traversing the membrane are hydroxide ions (OH-), which are produced by the cathode’s electrochemical oxygen reduction process (ORR). These OH- ions are carried to the anode, where the fuel (e.g., hydrogen, methanol, ethanol, etc.) reacts with electro-catalysts to produce water molecules for every single electron involved. Importantly, in AEMFCs, water is created simultaneously at the anode and used as a reactant at the cathode. To achieve effective performance of AEMFCs, the AEM must be able to facilitate the transfer of OH- ions while limiting fuel crossing. Simultaneously, it should hinder electron movement in order to prevent a circuit break, hence improving the overall efficiency and stability of the fuel cell system [115,125,126].

The application of alkaline direct alcohol fuel cells is limited by considerable hurdles in developing anion-exchange membranes that demonstrate both strong stability and efficient ion conductivity under fuel cell operating conditions. To solve this constraint, various PVA–polymeric materials are extensively tested to ensure their applicability. For instance, Higa et al. [127] developed a glyoxal (GA)-cross-linked anion-exchange membrane (AXM) using a new poly(vinyl alcohol)-b-poly(vinyl benzene trimethyl ammonium chloride) copolymer (PVA-b-PVBTAC). The improved membrane, cross-linked with 0.01 vol% GA, demonstrated a respectable power density of 99.6 mW cm^−2^ and a maximum current density of 247.8 mA cm^−2^. This performance outperformed a commercial AMX membrane with a power density of 15.2 mW cm^−2^ and a current density of 57.6 mA cm^−2^ under comparable conditions. The fuel cell, which ran at 60 °C and was fed with a 5.0 wt% methanol solution, proved the enhanced capabilities of the PVA-based multiblock copolymer. This copolymer, which has a microphase-separated morphology due to crystalline and noncrystalline regions within the polymer electrolyte membrane (PEM) structure, is thought to be advantageous for direct methanol alkaline fuel cell (DMAFC) applications that use highly concentrated methanol solutions.

Another type of polyvinyl alcohol (PVA)-based polymer used in the production of anion-exchange membranes (AXMs) is quaternized PVA copolymer (QPVA) [129]. Nanocomposite AXMs, which are made by combining quaternary amine-functionalized PVA with various nanomaterials such as graphene oxide (GO) [130], SiO_2_ [131], MoS_2_ [132], and Fe_3_O_4_-GO nanohybrid [133], have shown significant reductions in methanol permeability. To illustrate, Lin et al. [134] conducted a study where magnetite nanoparticles (Fe_3_O_4_) were synthesized on graphene oxide (Fe_3_O_4_-GO) and dispersed into a QPVA solution using ultrasonication. The resulting nanocomposite membrane was prepared through solution casting on a glass plate. During the drying process, a magnetic field was applied both on the top and bottom of the membrane to align Fe_3_O_4_-GO in QPVA along the through-plane direction. The nanocomposite QPVA/Fe_3_O_4_-GO membrane, thus obtained, exhibited impressive power densities of 200 mW cm^−2^ and 144.3 mW cm^−2^ when operated at 60 °C using 2 M methanol and 3 M ethanol, respectively. The observed low methanol permeability and high ionic conductivity of the membrane can be attributed to the gradient distribution of Fe_3_O_4_-GO in the QPVA matrix. This distribution creates Fe_3_O_4_-GO-rich regions at the surface and QPVA-rich regions at the middle, serving as a barrier for methanol while enhancing ionic conductivity. Table 5 summarizes the performance characteristics of various AEMs) designed for alkaline fuel cells. These membranes, composed of different polymer blends and cross-linking strategies, exhibit a range of ion-exchange capacities, tensile strengths, and alkaline stabilities.

## 3. Crafting PVA-Based Membranes: A Synthesis Overview

PVA is a notable example of a water-soluble, biodegradable polymer (Figure 4). PVA is not directly polymerized from the appropriate monomer, unlike most vinyl polymers. Rather, several indirect techniques are used, like alcoholysis of polyvinyl acetate (also referred to as saponification or hydrolysis) [35]. This conversion process can be facilitated by using potent acids or bases. As a polymeric material, PVA boasts exceptional properties contributing to its widespread utilization across diverse industrial applications. Remarkable attributes include its excellent film-forming capacity, structural flexibility, hydrophilicity, and biocompatibility [36,37]. Consequently, PVA finds extensive use in various industrial domains, including but not limited to the recovery of acids and valuable metals from waste solutions, membrane fuel cell applications, biological and biomedical applications, catalysis in organic and inorganic syntheses, as well as pervaporation separation of liquid mixtures [38,39,40,41]. Furthermore, several processes have been used to create porous membranes from PVA, including phase inversion and electrospinning [139], which is explained below in this review.

### 3.1. Phase Inversion Technique

**Figure 9:**
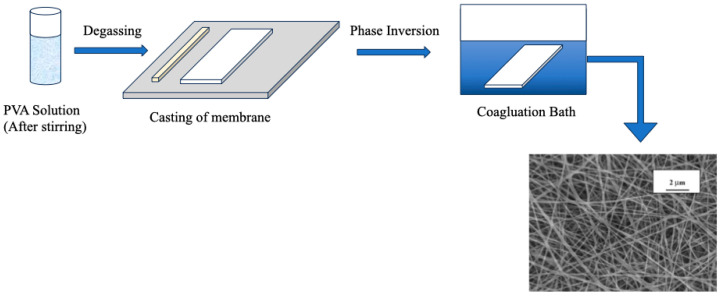
Schematic representation of phase inversion of PVA-based membrane. Redrawn from [143,144].

Phase inversion is the controlled separation of an originally homogeneous polymer solution into a polymer-rich and a polymer-lean phase (Figure 9). This separation is a vital step in membrane formation since the polymer-rich phase eventually forms the membrane’s solid skeleton, while the leftover polymer-lean phase creates pores. Structures formed by phase inversion frequently have sponge or finger-like macropores with sizes greater than 50 nm [140,141]. To illustrate, in the research carried out by Aiswarya et al. [142], a cation-exchange membrane based on PVA was manufactured for usage in direct methanol alkaline fuel cells (DMAFC) using the phase inversion approach combined with a chemical process. The study found that the PVA-based cation-exchange membrane had increased ionic conductivity as a result of the phase inversion technique paired with a chemical reaction.

In a separate study undertaken by Sidharthan et al. [145], polymer electrolyte membranes were created utilizing PVA and sulfosuccinic acid by the phase inversion approach. A variant was also made by adding montmorillonite (MMT) nanoparticles into PVA using sulfosuccinic acid. The study’s findings revealed that the addition of montmorillonite organoclay to the sulfonated PVA membrane had a major impact on its properties, as it demonstrated good proton conductivity and lower methanol permeability than the traditional Nafion membrane. In another study by Khalid et al. [146], a cross-linked poly(vinyl alcohol) (PVA)-based composite membrane was prepared using the phase inversion technique for ADMFCs. Titanium dioxide (TiO_2_) and carbon nanoparticles were incorporated into the PVA matrix to enhance mechanical and thermal properties. The membrane was further modified with maleic acid as a cross-linker. The cross-linked PVA-TiO_2_-C membrane demonstrated excellent thermal stability, high tensile strength (163 MPa), and low permeability (45,000 Ss/cm^3^). Additionally, the ionic conductivity was in the order of 10⁻^2^ S/cm, making it a promising candidate for ADMFC applications. Furthermore, Wibisono et al. [147] produced composite anion-exchange membranes (AEMs) using three different masses of colloidal silica (SiO_2_) as an inorganic filler, three mass ratios of polymer mixtures (with PVA as an inert polymer and PDDA as an active polymer), and three different solvents (H_2_O, DMSO, and DMF). The properties of the fabricated membranes were evaluated for use in reverse electrodialysis (RED) systems. The membranes produced with DMSO showed the best performance, with an area resistance (AR) of 1.69 kΩ·cm^2^, a swelling degree (SD) of 31.56%, and a fixed charge density (FCD) of 0.26 mmol/g H2O at the mixed mass ratio φ(PDDA/PVA)/SiO_2_ of AEM (0.528:0.4; DMSO). SiO_2_ was found to enhance the membrane matrix by forming -C(=O)-O-C- and -Si-O-Si- bonds and promoting quaternary ammonium bond formation. Increasing the ratio of the mixture improved AR and permselectivity. Additionally, using water as a solvent for preparing AEMs from the PVA/PDDA/SiO_2_ mixture demonstrated promising ion-exchange properties while being environmentally safe.

### 3.2. Electrospinning

**Figure 10:**
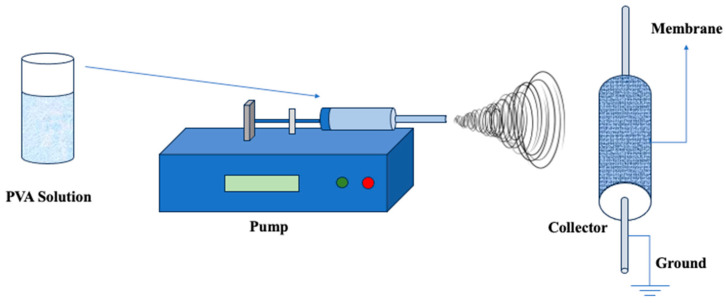
Illustration of the Electrospinning Process for PVA-based membrane fabrication. Redrawn from [152].

Electrospinning, an amalgamation of “electrostatic” and “spinning”, is a process used to create polymer nanofibers (Figure 10). This approach uses electrostatic forces to extract fibers from polymeric solutions, producing nanofibers with diameters ranging from 3 nm to 1 μm [148,149]. While electrospinning has not traditionally been a popular process for manufacturing porous materials, it has received considerable interest from academics in recent years. This renewed interest stems mostly from its transition into a potential production technology for a specific type of membrane known as electrospun nanofibrous membranes (ENMs). The careful selection of factors such as fiber diameter, stacking, and density enables the customization of ENMs’ asymmetric structure. This adjustment, in turn, gives certain properties to the membrane, such as high permeability, targeted liquid entry, and bubble point pressure. Although electrospinning was first used in the textile industry in the 1930s [150], its transformation into membrane technology [151], notably in the form of ENMs, represents a huge step forward with numerous applications in a variety of areas.

To demonstrate, Zizhou et al. [153] conducted a study on the electrospinning of Nafion^®^ nanofibers using PVA as a carrier polymer. Bead-free PVA/Nafion^®^ nanofibers were successfully generated using greater-molecular-weight PVA. Furthermore, two different stabilization procedures were used on PVA and PVA/Nafion^®^ nanofibers: BTCA cross-linking and thermal stabilization, followed by sulfonation of the PVA component. The study examined the membranes’ thermal stability, water resistance, methanol resistance, oxidative stability, and ion-exchange capacity. The results showed that the thermally stabilized PVA/Nafion^®^ nanofibrous membrane was resistant to water, methanol, and oxidative impacts.

In a different study led by Liao et al. [154], electrospun quaternized polyvinyl alcohol (Q-PVA) nanofibers were generated, and a potassium hydroxide (KOH)-doped nanofiber mat outperformed a dense Q-PVA film doped with KOH in terms of ionic conductivity. The composite, which included 5.98% electrospun QPVA nanofibers, showed a significant reduction in methanol permeability. The quasi-coaxial structure of the electrospun nanofibers was linked to the dual improvement in conductivity and suppression of methanol permeability. As a result, the Q-PVA composite evolved as a strong solid electrolyte, making alkaline fuel cells a realistic choice. In a direct methanol alkaline fuel cell operating at 60 °C, the electrospun Q-PVA composite demonstrated a peak power density of 54 mWcm^−2^, representing a 36.4% increase over a cell using a pristine Q-PVA film. Moreover, Barlybayeva et al. [155] developed a novel anion-exchange membrane (AEM) using a deep eutectic solvent (DES)-supported polymer-based approach. This method involved impregnating a cross-linked PVA-based nanofiber mat with DES through a soaking process. The PVA-based nanofibers were produced via electrospinning and subsequently cross-linked with glutaraldehyde (GA) in concentrations ranging from 4 wt.% to 8 wt.%. The DES was synthesized by combining choline chloride (ChCl) and ethylene glycol (EG) at a molar ratio of 1:3. The resulting AEM, modified with DES (DES3@PVA4), demonstrated impressive electrochemical properties, including a conductivity of 0.66 mS/cm at room temperature and 1.05 mS/cm at 60 °C. Furthermore, the membrane exhibited no swelling and significantly improved elongation at break, increasing from 0.24% to 1.08%. These findings suggest that the DES-supported PVA-based composite membrane possesses high hydroxide conductivity, flexibility, and mechanical stability in a fully hydrated state, making it a promising candidate for AFC applications. In a different study by Gómez et al. [156], a binary polymeric blend consisting of CS and PVA in an 80:20 ratio was developed to create a solid polymer electrolyte for potential use in proton- or anion-exchange electrochemical cells. Using a 6% m/m solution, membranes were fabricated through electrospinning, and the effect of incorporating titanium oxide (TiO_2_) nanoparticles at concentrations ranging from 1000 to 50,000 ppm on the physicochemical properties of the material was investigated. SEM micrographs revealed that the nanofibers had diameters close to 100 nm. The inclusion of TiO_2_ nanoparticles impacted moisture absorption, resulting in hydrophobic behavior at higher concentrations (2% absorption) and higher absorption values (up to 13%) at lower concentrations. Consequently, thermogravimetric analysis (TGA) indicated reduced dehydration in composites with 1000 ppm TiO2, while differential scanning calorimetry (DSC) showed that the nanoparticles did not significantly alter the thermal transition temperature (Tm) of the composite. The incorporation of TiO_2_ shifted the glass transition temperature (Tg) from 44 °C to 37 °C, suggesting improved ionic conductivity at room temperature. Complex impedance spectroscopy revealed that the activation energy of the material decreased with the addition of 1000 ppm TiO_2_. Additionally, doping the membranes with a 1 M KOH solution transformed their fibrous structure into a porous cork-like configuration. Future research aims to explore the use of these electrospun membranes in developing composites to validate the energy efficiency of the new solid polymer electrolyte.

## 4. Key Influencing Factors on the Performance of PVA-Based Membranes in Fuel Cells

The efficacy of PVA-based membranes in fuel cell applications is determined by various parameters that substantially impact the membrane’s mechanical strength, durability, and processability. These elements, which are critical in defining the membrane’s overall performance, include a wide range of concerns, including both material properties and processing parameters. Material qualities, reinforcing tactics, and fabrication procedures all impact the membrane’s mechanical strength, which is a crucial determinant of its structural integrity [157]. Similarly, the membrane’s endurance is determined by the environmental conditions encountered during fuel cell operation, emphasizing the need for materials that are more resilient to chemical, thermal, and mechanical stresses. Furthermore, the processability of the membrane, which is critical for its manufacturability and simplicity of integration into fuel cell systems, is influenced by material selection, fabrication techniques, and overall compatibility with industrial-scale production processes. Thus, a thorough understanding of these multiple aspects is required to maximize the performance of PVA-based membranes in fuel cell applications, enabling improvements in mechanical robustness, long-term durability, and overall manufacturability.

Scientifically, PVA inherently lacks fixed charges and tends to swell easily in aqueous environments, compromising its proton conductivity and mechanical strength in membrane applications. To address these limitations and enhance the suitability of PVA membranes for DMFCs, studies discussing the coming modifications are imperative [158,159,160]. Enhancing proton conductivity in PVA membranes often involves combining PVA with other materials possessing acid groups. Examples include poly(2-acrylamido-2-methyl-1-propanesulfonic acid) (PAMPS) [161,162], poly(styrene sulfonic acid) [163,164], phosphotungstic acid (PWA) [165], or cross-linking with poly(styrene sulfonic acid-co-maleic acid) [166,167], sulfosuccinic acid (SSA) [168], and poly(acrylic acid) [169]. In addition, an alternate strategy to improving PVA membrane characteristics is to fabricate organic–inorganic composites [170,171,172]. Adding inorganic particles such as Al_2_O_3_ and SiO_2_ to the polymer matrix has a multidimensional effect on membrane properties. This inclusion prevents methanol crossing and improves conductivity and mechanical qualities [173]. Despite these potential advantages, producing organic–inorganic composites presents obstacles in guaranteeing the uniform dispersion of inorganic particles inside the polymer matrix [174,175]. In this respect, the impact factors include the effectiveness of blocking methanol crossover, the improvement in conductivity and mechanical properties, and the inherent difficulties of ensuring uniform dispersion of inorganic particles throughout the composite construction process. Table 6 illustrates the common impact factors on PVA-based membranes in fuel cell industry.

## 5. Enhancements and Modifications of PVA-Based Membranes for Fuel Cells

**Figure 11:**
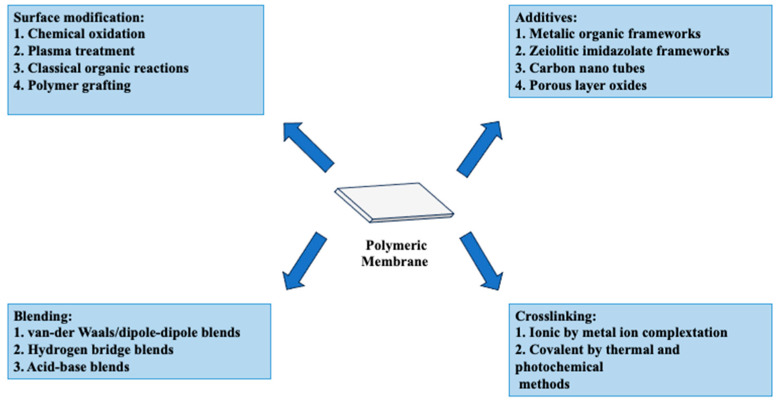
Approaches to enhance the performance of PVA membranes in fuel cell technology. Extracted from [157,176,188,189,190,191,192].

The inherent high penetration selectivity of PEMs to water over alcohol prompted research into PVA’s application in DMFC contexts. This feature is thought to provide superior methanol barrier qualities compared to Nafion membranes. Furthermore, the hydroxyl groups in PVA may interact with other host polymers, enhancing the overall stability of the blended composite system (Figure 11). Maiti et al. [179] comprehensively assessed over 40 relevant studies on modified PVA for DMFC applications. PVA-based membranes are increasing in competitiveness over Nafion membranes, particularly due to two key characteristics: proton conductivity and methanol.

### 5.1. Enhancing PVA Membrane Performance through Cross-Linking/Copolymerization

**Figure 12:**
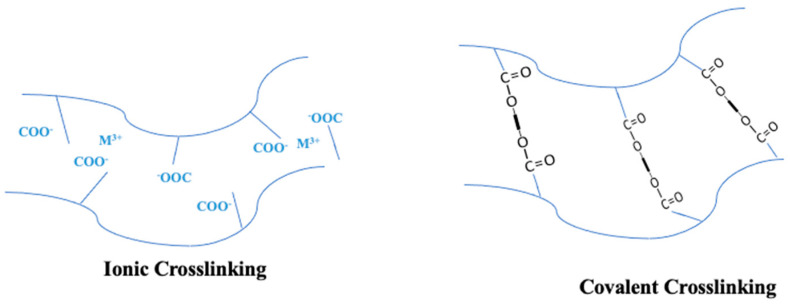
Cross-linking Methods in Polymers with Carboxyl Groups: Ionic Cross-linking via Metal Ion Complexation and Covalent Cross-linking via Thermal or Photochemical Processes. Reproduced from [193].

While PVA membranes have superb resistance to chemicals and film-forming properties, there is ongoing research to improve their stability. Numerous studies are underway to investigate techniques for enhancing the stability of this material. One such strategy is to cross-link the PVA polymer via chemical reactions with particular solutions. However, the general cross-linking of polymers (as seen in Figure 12) is instrumental in conferring low solubility, achieved through the covalent bonding of only one type of monomer/molecule that impedes the easy sliding of polymer molecules [193]. Several cross-linking strategies have been studied for PVA membranes, including the use of cross-linking chemicals such as glutaraldehyde [194,195,196], formaldehyde [197], sulfur-succinic acid [95], heat treatment [198], freezing [199], and UV radiation [200]. Similarly, graft copolymerization is one of the most frequent polymer modification techniques used to create hybrid polymers from natural and synthetic sources [201]. It amalgamates the characteristics of two or more polymers into a cohesive structure, distinguishing it from cross-linking [202].

Natural polymers in their inherent state often prove unsuitable due to heightened swelling and inadequate stability in biological surroundings. Graft polymerization serves as a valuable research avenue for altering these attributes, thereby yielding innovative polymeric materials endowed with hybrid properties [203]. Numerous studies have investigated the graft copolymerization of PVA with various hydrophilic and hydrophobic monomers in order to improve physical, mechanical, or biological properties [204,205].

As for cross-linking, Das et al. [167] created an alternative to the Nafion membrane for microbial fuel cells (MFCs) using a membrane made of PVA cross-linked with glutaraldehyde (GA). This low-cost membrane showed improved performance in MFCs after the cross-linking process, which resulted in a reduction in hydroxyl groups, as evidenced by controlled water uptake and swelling ratio, as well as improved thermo-mechanical stability via the formation of acetal rings and ether linkages. The cross-linked membrane produced significantly higher power density than standard MFCs fed with household wastewater, reaching a maximum of 158.28 mW/m^2^. In another study, Kulasekaran et al. [182] crafted polymer electrolyte membranes by chemically cross-linking PVA and sulfonated poly(ether sulfone). The polymerization synthesis used three different monomers: bisphenol A, phenolphthalein, and 4,40-dichlorodiphenyl sulfone. The resulting blended membrane showed a high ion-exchange capacity and percentage water uptake. At 30 °C, the membrane had a higher proton conductivity (0.0367 S cm⁻^1^) than pure PVA (0.0259 S cm⁻^1^). The findings align with those of Gupta et al. [206], who observed that chemical cross-linking enhances proton conductivity and augments mechanical strength. In their study, chemically cross-linked PVA-based KOH-doped alkaline membranes were developed for application in ADEFC.

In reference to copolymerization, Awad et al. [207] successfully constructed a proton-exchange membrane, specifically a polyvinyl alcohol/silicon nanoparticles (PVA/SiO_2_) composite, using gamma radiation to promote graft copolymerization of styrene monomer on a polyvinyl alcohol screen. The ion-exchange capacity (IEC) indicated improvement with an increase in the degree of grafting, followed by a minor reduction due to the homo-polymerization of styrene increasing with greater doses of gamma irradiation. Proton conductivity increased by up to 30% depending on the degree of grafting before stabilization. Additionally, Danwanichakul et al. [208] developed a membrane made of chitosan-grafted poly(vinyl alcohol)/poly(vinyl alcohol) (CS-g-PVA/PVA) and studied its water and methanol transport qualities, mechanical properties, and ionic conductivity. The cross-linked CS-g-PVA/PVA membrane has an ionic conductivity of 4.37 mS cm^−1^ and a methanol permeability (Ps) of $1.8\times 10^{-7}$ cm^2^ s^−1^, which yielded a selectivity σPs of 23.95 mS·s·cm^−3^. Notably, the conductivity of the cross-linked CS-g-PVA/PVA membrane improved significantly when saturated with a methanol solution with a concentration more than 20%. Furthermore, Aoshima et al. [209] introduced an innovative approach to synthesize PVA graft copolymers through living cationic polymerization, employing a novel coupling reagent known as partially hydrolyzed poly(vinyl acetate) (PVAc-OH). This method resulted in enhanced solubility compared to pure PVA. The grafted PVA demonstrated solubility in water, dimethyl sulfoxide (DMSO), and various organic solvents, such as toluene and chloroform (CHCl_3_). Films derived from PVA-graft-poly(2-methoxyethyl vinyl ether) (poly(MOVE)) exhibited translucency and toughness, while a nearly transparent film was obtained from the combination of PVA and PVA-graft-poly(MOVE).

### 5.2. Enhancing PVA Membrane Performance through Blending

The blending of polymers produces a polymer blend or mixture, in which at least two polymers are blended to generate a unique substance with distinct physical properties [210]. This method, as opposed to using a single polymer, provides a more simple and appealing method for improving many properties, including biological, mechanical, and deteriorating elements. For environmentally sustainable manufacture, a blend of synthetic and natural polymers is preferred where this arises from the flexibility provided by combining varying proportions of these components, which allows for the development of a wide range of polymer properties [211,212]. This preference is due to the fact that synthetic polymers, unlike their natural counterparts, frequently have adjustable functional groups, allowing for the attainment of desired mechanical and chemical properties. Overall, achieving a homogenous mixture through proper mixing has a significant impact on the blending process. This feature emphasizes the significance of obtaining molecular-level miscibility in polymer blends, which directly effects the overall blending behavior and the consequent material qualities [213].

In several investigations spanning different applications [214,215,216], it has been discovered that blending PVA and chitosan results in poor miscibility. These investigations have revealed that weak hydrogen-bonding interactions are responsible for this outcome, and the degree of hydrolysis in PVA has a substantial impact on the blend’s performance by culminating in low miscibility levels. In the context of fuel cells, Qiao et al. [217] created chemically cross-linked polymer blend membranes made of poly(vinyl alcohol) (PVA), 2-acrylamido-2-methyl-1-propanesulfonic acid (PAMPS), and poly(vinylpyrrolidone) (PVP). As the cross-linking time increased, membrane swelling decreased, resulting in better mechanical characteristics and a modest decrease in proton conductivity due to reduced water absorption. The membranes had a proton conductivity of 0.088 S cm^−1^ and an ion-exchange capacity (IEC) of 1.63 mequiv g^−1^ at 25 ± 2 °C. The polymer composition was PVA-PAMPS-PVP with a mass ratio of 1:1:0.5. The methanol permeability was measured at 6.1 × 10^−7^ cm^2^/s. The membranes demonstrated relatively strong oxidative durability, with minimal weight loss after prolonged exposure.

Gouda et al. [218] recently developed a binary polymer blend employing cost-effective and environmentally acceptable polymers—polyethylene oxide (PEO) and polyvinyl alcohol (PVA)—incorporating phosphated titanium oxide nanotubes (PO_4_TiO_2_). Adding PO_4_TiO_2_ improved physicochemical parameters, including increased oxidative stability and tensile strength, while reducing borohydride permeability, water uptake, and swelling ratio. Notably, the membranes exhibited increased ionic conductivity and power density, reaching 28 mS cm^−1^ and 72 mWcm^−2^, respectively, for the membrane with 3 wt% of PO_4_TiO_2_. This membrane demonstrated approximately 99% oxidative stability and a tensile strength of 40.3 MPa. Additionally, the membrane displayed high selectivity and showed promise as a proton-exchange membrane, suggesting its potential application in developing environmentally friendly and cost-effective direct borohydride fuel cells (DBFCs).

In another recent study conducted by Sidharthan et al. [219], a novel approach was undertaken to develop environmentally friendly sodium ion-conducting membranes based on polyvinyl alcohol (PVA). The membrane fabrication involved using a green nonsolvent coagulation bath, which consisted of sodium hydroxide and sodium sulfate. This innovative membrane, denoted as sulfonated polyvinyl alcohol (SPVA)–montmorillonite clay (MMT), was specifically designed for operation in an alkaline medium. This development aimed to address the limitations associated with proton-exchange membranes functioning in acidic environments and anion-exchange membranes employed in alkaline conditions. The SPVA-MMT membrane exhibited a notable ionic conductivity of 0.0751 S cm⁻^1^, attributed to the presence of inorganic salts in the coagulation bath and the cation-exchange capacity of the clays. The SPVA-MMT membrane demonstrated promising performance in DMFC, achieving a peak power density of 7.51 mW cm⁻^2^ under ambient conditions. This research signifies a significant step towards sustainable and effective membrane materials for alkaline DMFC applications.

## 6. Challenges Associated with PVA-Based Membranes for Fuel Cells and Future Aspects

The use of polyvinyl alcohol (PVA) membranes in fuel cells comes with a set of challenges that researchers are actively working to overcome. While PVA membranes boast excellent resistance to chemicals and form strong films, there is a constant push to enhance their stability, a critical factor for sustained performance. Furthermore, the development of PEMs for fuel cell applications requires careful consideration of particular parameters. These critical requirements include but are not limited to (a) cost effectiveness, (b) strong thermal and chemical stability, (c) improved mechanical strength, (d) increased proton conductivity, and (e) minimal methanol crossover. The utilization of water-soluble polymer blends in crafting PEMs is considered a more environmentally friendly and cost-effective approach to membrane preparation. This is attributed to the minimized use of harmful organic solvents, simplifying the fabrication process. However, for these PEMs to excel in practical applications, urgent attention is required to enhance long-term and thermal stabilities [220]. Addressing these aspects will be pivotal in unlocking the full potential of water-soluble polymer blends for PEMs, ensuring their practicality and efficacy across various applications.

In the field of DMFC applications, achieving high ionic conductivity is a critical condition for membrane performance. When designing innovative PVA-based polymers, exploring ways to successfully minimize water content while maintaining commendable ionic conductivities is critical. The developmental processes should incorporate inorganic components into the organic matrix in a way that makes the best use of elementary functions in a limited space. This technique seeks to synergistically improve the potential and qualities of both inorganic and organic components, thereby optimizing their complementary capabilities [179]. Contrary to the advancements in improving properties like chemical, thermal, and mechanical aspects through various modification methods for PVA-based PEM, a significant challenge remains in the relatively low power densities observed in DMFC applications. The recorded power densities range from 1 to 24 mWcm^−2^ within the temperature range of 25 to 80 °C [221]. While modifications show promise in enhancing overall PEM characteristics, uncertainties persist regarding the arrangement of the polymer matrix following these modifications. Henceforth, considerable research endeavors are currently underway to tackle these obstacles, the intricacies of which are detailed below. Based on the literature reviewed, it is evident that polyvinyl alcohol (PVA) is emerging as a promising candidate for fuel cell applications, offering an alternative to traditional membranes. Membrane technology is crucial in addressing the increasing global demand for electricity and water. PVA, characterized by its numerous hydroxyl groups and robust hydrogen bonding with water, exhibits remarkable resistance to methanol, thus garnering significant attention in contemporary fuel cell research endeavors. Thereupon, the emphasis on DMFC technology underscores efforts to improve methanol barrier characteristics while lowering manufacturing costs. Cross-linking, copolymerization, and blending with other polymers are being investigated as ways to address intrinsic constraints, such as PVA membranes’ low ionic conductivity. These improvements seek to achieve a compromise between better performance and cost effectiveness, both of which are critical for wider adoption of fuel cell technologies. On the other hand, PVA’s use in AEMs for alkaline fuel cells expands its versatility, where choosing appropriate polymers for AEMs becomes critical, emphasizing the need for membranes with high hydroxide ion conductivity, stability, flexibility, and cost effectiveness. As research advances, PVA-based membranes demonstrate potential for delivering long-term energy generation options.

In essence, PVA’s path in fuel cell technologies is woven through a tapestry of breakthroughs, difficulties, and goals, each contributing to the continued evolution of membranes in the search of efficient and sustainable energy conversion.

Future advances in modifying PVA membranes are expected to offer substantial breakthroughs, utilizing a mix of approaches to overcome fundamental tradeoff difficulties. The research of Chang et al. [222] exhibited this strategy by demonstrating the fabrication of a PVA/sulfonated polyhedral oligosilsesquioxane (sPOSS) hybrid membrane, in which this novel membrane was created using a simple technique that involved combining PVA and sPOSS solutions and then cross-linking with ethylenediaminetetraacetic dianhydride (EDTAD). The study found that adding sPOSS into the membrane significantly increases proton conductivity, peaking at 50 wt.% (0.042 S/cm at 25 °C). Furthermore, the hybrid membrane has higher thermal stability than pure PVA, indicating the intriguing future potential of mixed modification schemes for increased performance.

In line with this strategic approach, other researchers have used similar strategies to solve the different difficulties associated with PVA membranes in fuel cell applications. For example, Gajbhiye et al. [223] worked on developing a PVA-g-PMA hybrid membrane for usage in DMFC. Maleic anhydride (MA) was grafted onto PVA, utilizing both ionic and chemical methods, including potassium persulfate. The produced PVA-g-PMA was then mixed with 3-Amino-4-[3-(triethylammonium sulfonato)phenyl amino]phenylene hydrochloride. The new membrane had higher fuel cell power (34.72 mW/cm^2^) and current density (62.11 mA/cm^2^) than the Nafion 117 membrane, demonstrating the effectiveness of this modification method in improving performance. In a separate investigation, Murmu et al. [224] developed SPEEK–PVA–silica hybrid membranes using a solution casting approach. The study evaluated water and methanol absorption capacity, IEC, degree of sulfonation (DS), hydration number (λ), void volume fraction (%), methanol permeability, and proton conductivity. The results showed that as the silica content grew, so did the polymer membrane’s void volume fraction and density, resulting in a lower glass transition temperature. At 30 °C, the SPS-3 membrane had the highest proton conductivity (3.8 × 10^−2^ S/cm), surpassing other results in the study. Furthermore, the silica-filled polymer membrane’s increased conductivity at medium to high temperatures was attributable to a strong contact between silica and water, which reduced water evaporation losses and improved bounding. Furthermore, the reduced methanol permeability and higher membrane selectivity indicated the silica-filled polymer’s outstanding methanol blocking capacity, which provides benefits for the design of direct methanol fuel cells.

Furthermore, various investigations have looked into the modification of PVA membranes for fuel cell applications. These studies have explored a wide range of strategies targeted at improving membrane performance. Among the approaches that have been explored are chemical modifications [162,225], composite blending [226,227,228], thermal cross-linking [229,230,231], and the incorporation of nanocomposites [191,232]. These diverse approaches demonstrate the substantial efforts to improve the properties and capacities of PVA membranes in the context of fuel cell technology.

## 7. Conclusions

The quest for efficient and sustainable energy conversion in fuel cell technology has sparked substantial study, with a particular emphasis on improving the performance of PVA membranes. These membranes play a vital role in fuel cells by influencing proton conductivity, thermal stability, and overall efficiency. Future research endeavors should prioritize enhancing membrane performance by exploring techniques that positively influence the properties and capacities of PVA membranes. This includes delving into innovative approaches such as advanced chemical alterations, novel composite blending methods, precise thermal cross-linking techniques, and the incorporation of cutting-edge nanocomposites. By focusing on these aspects, researchers can deepen their understanding and further advance the capabilities of PVA membranes for fuel cell applications. The combined efforts shown in these investigations demonstrate various modification strategies for tailoring PVA membranes for optimal fuel cell performance. As the research landscape evolves, these unique approaches pave the way for developing efficient and sustainable fuel cell technologies, which hold promise for future energy needs. Moreover, researchers need to consider both the economic and environmental aspects during the development and optimization of PVA membranes. Striking a balance between cost effectiveness and sustainability is crucial to ensure the utmost efficiency and applicability of fuel cell technologies. By encompassing these considerations into their investigations, researchers can contribute to realizing efficient and sustainable fuel cell technologies that fulfill future energy demands.

## Figures and Tables

**Table 2:** A detailed overview on the critical properties of PVA.

Property	Description	References
**Chemical Nature**	Copolymer consisting of hydroxyl and acetyl units resulting from incomplete hydrolysis of poly(vinyl acetate).	[47]
**Physical State**	Odorless, tasteless, water-soluble polymer.	[34,48]
**Crystallinity**	Semi-crystalline.	[49]
**Thermal Stability**	Thermostable with melting points around 220–230 °C for fully hydrolyzed grades and 180–190 °C for partially hydrolyzed ones.	[34,50]
**Biocompatibility**	Non-toxic, biocompatible, and biodegradable in aerobic and anaerobic environments.	[51,52]
**Optical Properties**	Exhibits remarkable optical properties.	[53,54]
**Charge Storage Ability and Dielectric Strength**	Exceptional charge storage ability and high dielectric strength.	[55]
**Solubility**	Soluble in polar solvents like N-methyl-2-pyrrolidone, dimethyl sulfoxide, glycerol (hot), and others. Not soluble in lower alcohols, ketones, esters, and others.	[45,56]
**Crystal Structure**	Monoclinic crystal cell structure.	[57,58,59]

**Table 4:** Comparison of Performance Metrics for PVA–Proton-Conducting and Methanol Barrier Membranes in DMFCs.

Membrane Composition	Methanol Permeability (cm ^2^/s)	Proton Conductivity (S/cm)	Ion Exchange Capacity (meq/g)	Remarks
Pure PVA	3.57 × 10^−7^	3.06 × 10^−3^	0.462	Pure PVA membrane with notable methanol barrier capability
PVA with sulfoacetic acid sodium salt (10 wt.%)	5.21 × 10^−9^	0.57 × 10^−3^	0.092	Improved methanol resistivity and moderate proton conductivity through chemical cross-linking
Phosphorylated PVA and grafted cellulose acetate (CA)	1.08 × 10^−10^	0.035–0.05	2.1	Low methanol permeability and enhanced proton conductivity and IEC with O-phosphoric acid (OPA)
PVA/PAA/SSA hybrid membrane	10^−8^ to 10^−7^	10^−3^ to 10^−2^	0.46–1.58	Cross-linked with sulfonic acid groups and silica particles to reduce methanol permeability while maintaining proton conductivity
PVA/PSSA_MA/Silica (20% TEOS)	5.55 × 10^−7^	0.026	0.95	High proton conductivity and low methanol permeability, with TEOS aiding in the formation of proton- conduction pathways
PVA/PSSA-MA (1:9 ratio)	2.53 × 10^−7^	0.1	3.41	Highly charged membranes with excellent proton addition

**Table 5:** Performance Characteristics of AEMs for Alkaline Fuel Cells.

Membrane Composition	Ion-Exchange Capacity (mmol/g)	Tensile Strength (MPa)	Alkaline Stability	Notes	Ref.
PVA/Poly(2,6-dimethyl-1,4-phenylene oxide) (PPO)	1.46	30.8	Retains 66.5% conductivity after 1000 h in 1 M NaOH at 80 °C	High mechanical strength, relatively low swelling ratio, and peak power density of 78 mW/cm^2^	[135]
PVA/qPPO (30% qPPO)	1.28	126 (dry). 41 (wet)	Minimal permselectivity change (0.92 to 0.90) over 10 days in 6 M KOH at room temperature	High OH^−^ conductivity, stable in alkaline water electrolysis, and retains permselectivity	[136]
Brominated PPO/N-methylmorpholine (NMM-18)	1.74	22.2	More stable than QPPPO membrane in 2 M NaOH at room temperature	High hydrophilicity, low membrane area resistance (1.5 Ω·cm^2^), and good electrodialytic performance	[137]
CMPSP with high DCM (1.95)	1.65	0.07	9–13% loss in IEC after 48 h in 5.0 M KOH at 50 °C	High ion-exchange capacity, improved hydrophilicity, good desalination rate (94.5%), and low energy consumption (7.5 kWh/kg)	[138]

**Table 6:** Factors influencing the performance of polyvinyl alcohol membranes in fuel cell applications.

Impact Factors	Description	References
**Mechanical Strength**	Material qualities and reinforcing tactics determine mechanical strength	[176,177]
	Reinforcement methods and fabrication procedures directly impact structural integrity	
**Durability**	Environmental conditions during fuel cell operation affect membrane endurance	[178,179]
	Resilience to chemical, thermal, and mechanical stresses is crucial for prolonged functionality	
**Processability**	Influenced by material selection and fabrication techniques	[180,181]
	Compatibility with industrial-scale production processes is a key consideration	
**Proton Conductivity and Swelling**	PVA’s inherent lack of fixed charges and propensity to swell compromise proton conductivity and mechanical strength	[182,183]
**Material Modifications**	Enhancing proton conductivity involves combining PVA with materials possessing acid groups	[184,185]
**Organic–Inorganic Composites**	Examples include PAMPS, poly(styrene sulfonic acid), phosphor tungstic acid, or cross-linking with other substances	[186,187]
	Addition of inorganic particles (e.g., AI2_2_O_3_ and SiO_2_) prevents methanol crossover and improves conductivity and mechanical qualities	

## Data Availability

Not applicable.
